# Assessment of Higher Ordered Thinking in Medical Education: Multiple Choice Questions and Modified Essay Questions

**DOI:** 10.15694/mep.2018.0000128.1

**Published:** 2018-06-12

**Authors:** Arslaan Javaeed

**Affiliations:** 1 University of Ottawa

**Keywords:** multiple choice questions, modified essay questions, assessment higher ordered thinking

## Abstract

This article was migrated. The article was marked as recommended.

**Background:** Multiple choice questions and Modified Essay Questions are two widely used methods of assessment in medical education. There is a lack of substantial evidence whether both forms of questions can assess higher ordered thinking or not.

**Objective:** The objective of this paper is to assess the ability of a well-constructed Multiple-Choice Question (MCQ) to assess higher ordered thinking skills as compared to a Modified Essay Questions (MEQ) in medical education.

**Methods:** The medical education literature was searched for articles related to comparison between multiple choice questions and modified essay questions, looking for credible evidence for using multiple choice questions for assessment of higher ordered thinking.

**Results and Conclusion**: A well-structured MCQ has the capacity to assess higher ordered thinking and because of many other advantages that this format offers. Multiple choice questions should be considered as a preferable choice in undergraduate medical education as literature shows that different levels of Bloom’s taxonomy can be assessed by this assessment format and its use for assessing only lower ordered thinking i.e. recall of knowledge, is not very convincing.

## Introduction

**What is Higher ordered thinking?**

Higher ordered thinking is usually defined in reference to the cognitive domain of Bloom’s Taxonomy ( Fig I). First two levels, which are considered as lower ordered thinking, include remembering and understanding whereas rest of the four levels, constituting higher ordered thinking, include application, analysis, evaluation, and creation of knowledge in an ascending order ( Anderson, Lorin, Krathwohll, & Bloom., 2001).

**Figure 1.**
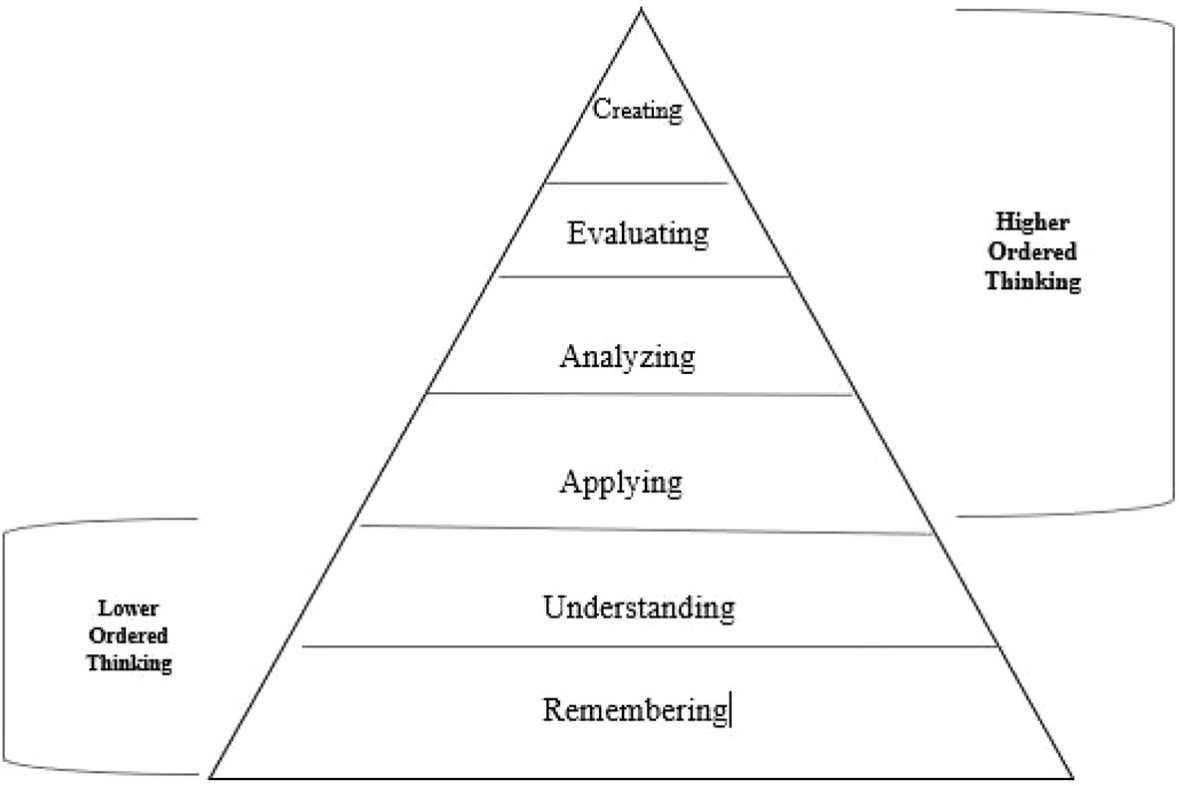
Levels of thinking in revised Bloom's Taxonomy.

### 1. Bloom’s Taxonomy to Revised Bloom’s Taxonomy

Bloom’s Taxonomy described and published in 1956 had permeated teaching for almost 45 years before it was modified in 2001. In Table 1, these existing taxonomies of cognition are discussed.

**Table 1.**
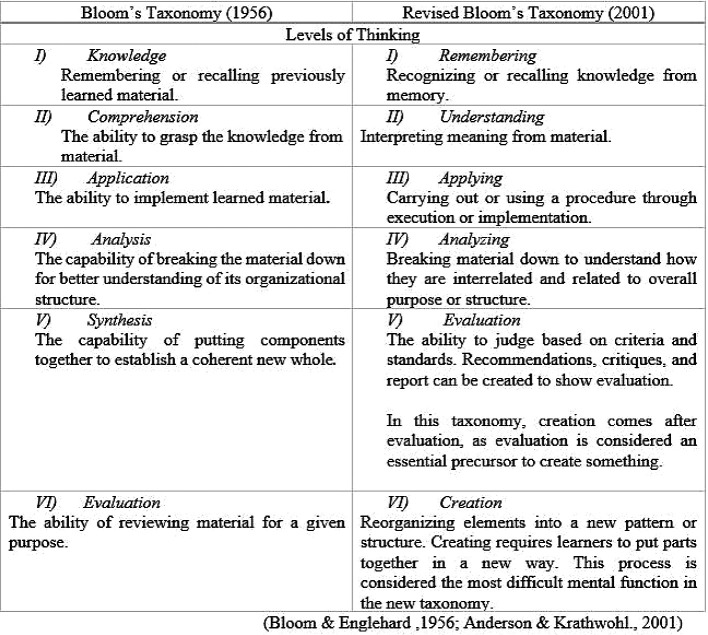
Comparison between Bloom's and revised Bloom's Taxonomy

### 2. MCQs and MEQ in Medical Education

There has been a considerable revision in undergraduate medical curriculum particularly in the assessment and teaching methodology. Written tests are an essential component of medical education. Objectivity is gradually replacing subjective assessment. Long essay type questions have been substituted by MEQs and MCQs. There is an ongoing debate on which assessment format should be administered to test higher ordered thinking ( Mehta, Bhandari, Sharma, & Kaur, 2016).

Assessment formats are mere tools and their usefulness can be hampered by their poor design, proficiency of its user, deliberate abuse and unintentional misuse (Tom Kubiszyn, 2013). To establish usefulness of a particular assessment format, the following five criteria should be considered: (1) reliability (2) validity (3) influence on future thinking and practice (4) suitability to learners and teachers (5) expenses (to the individual student and institution) ( Vleuten, 1996). Reliability is the degree to which a measurement produces consistent results ( Salkind, 2006) and validity means that how well the test measures which it intends to measure ( American Educational Research Association, American Psychological Association, & National Council on Measurement in Education, 2014).

## Discussion

MCQs are extensively used for assessment in medical education owing to their ability to offer a broad range of examination items that incorporate several subject areas. They can be managed in a relatively short period of time. Moreover, they can be marked by a machine which makes the examination standardized ( Epstein, 2007). There is a general perception that MCQs emphasize on knowledge recall i.e. Level I of revised Bloom’s Taxonomy and MEQs are capable of testing higher ordered thinking. The criticism against MCQs is basically due to its poor construction rather than the format itself. A study reveals that in assessing cognitive skills, MCQs significantly correlate with MEQs when their assessment’s content is matched ( Palmer & Devitt, 2007).

The modified essay question is a compromise between an essay and a multiple-choice question. Although it is well documented that a well-constructed MEQ tests higher ordered thinking, it is appropriate to ask if MEQs in undergraduate medical education are well-constructed and test higher ordered thinking. Mostly MEQs only test factual knowledge i.e. lower ordered thinking and at the same time risk significant variation in standards of marking as they are usually hand-marked ( Palmer & Devitt, 2007), rendering it unreasonable as an assessment format for testing a large number of students ( Sam, Hameed, Harris, & Meeran, 2016). Besides, it is a difficult task to construct an MEQ capable of testing higher ordered thinking in students and is more frequently associated with item writing flaws ( Khan & Aljarallah, 2011).

In contrast to MEQ, MCQs are suitable for testing a large number of students as they are machine scored ( Morrison & Walsh Free, 2001). Research shows that multiple choice questions assessing comprehension, application and analysis have been identified. This suggests that the ability of MCQs to assess higher ordered thinking is persistently undervalued and indicates that MCQs have the potential to assess higher ordered thinking ( Scully, 2017). Examples of multiple choice question assessing higher ordered thinking i.e. application ( Table 2), analysis ( Table 3) and evaluation levels ( Table 4, 5 and 6) are as follows:

**Table 2.**
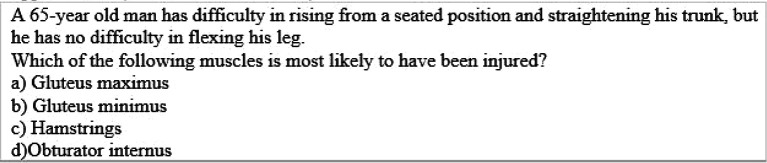
Example of Multiple Choice Question assessing higher ordered thinking i.e. Level III “Application” (Case & Swansin, 2002)

**Table 3.**
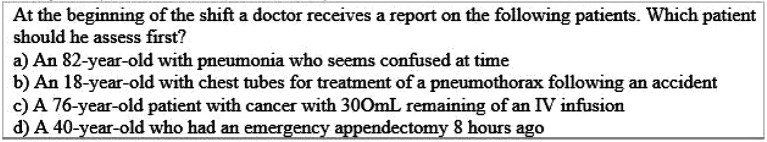
Example of Multiple Choice Question assessing higher ordered thinking i.e. Level IV “Analysis” (Oermann & Gaberson, 2009)

**Table 4.**
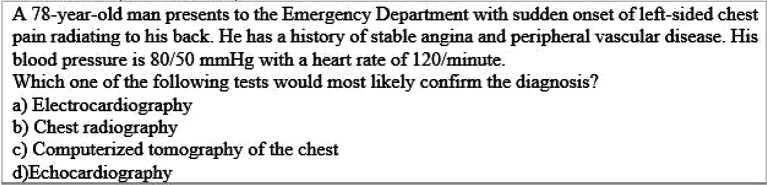
Example of Multiple Choice Question assessing higher ordered thinking i.e. Level V “Evaluation” (Touchie, 2010)

**Table 5.**
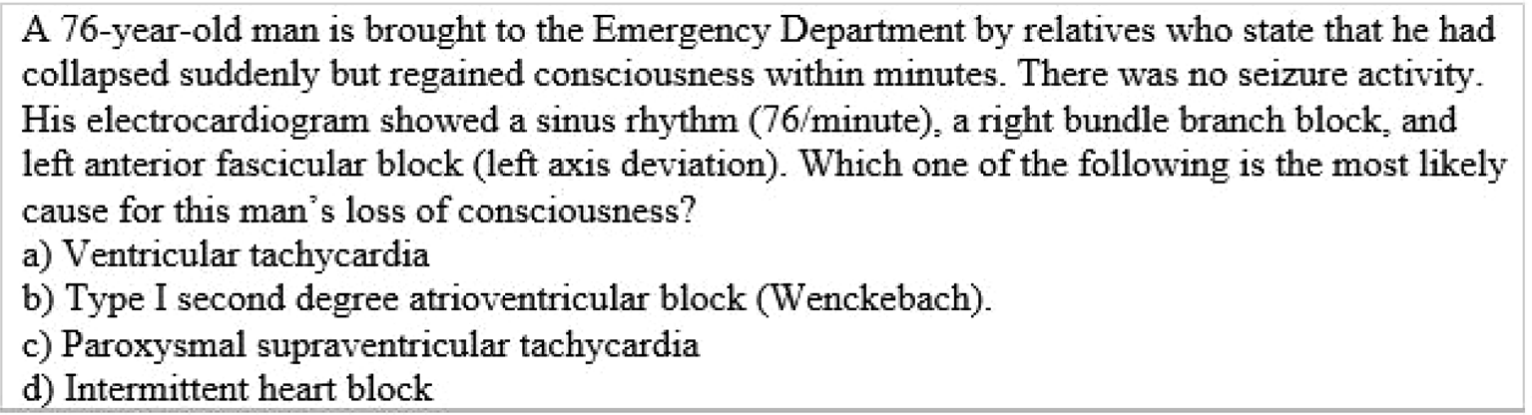
Example of Multiple Choice Question assessing higher ordered thinking i.e. Level V “Evaluation” (Touchie, 2010)

**Table 6.**
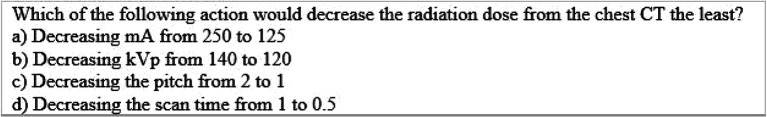
Example of Multiple Choice Question assessing higher ordered thinking i.e. Level IV “Evaluation” (Collins, 2006)

For a number of purposes, the significance of measuring higher ordered thinking is well renowned in medical education. It has been debated that multiple choice format is useful because it is reliable, objective, unbiased and efficient, cost-effective in nature but incapable of measuring higher ordered thinking. This is not true. A more correct declaration would be that MCQs measuring higher ordered thinking are rarely constructed and MCQs assessing lower ordered thinking are over-presented. One of the reasons of this over presentation is that the most item writers are not formally trained. This emphasizes that format itself is not limited to the assessment of lower ordered thinking. In undergraduate medical education, a well-constructed MCQ can easily assess a student’s ability to apply, evaluate and judge medical education knowledge ( Vanderbilt, Feldman, & Wood, 2013). Nevertheless, writing MCQs capable of assessing higher ordered thinking are challenging ( Bridge, Musial, Frank, Thomas, & Sawilowsky, 2003) but can be developed by following certain guidelines, especially ensuring that item writers are competent in their fields ( Haladyna, & Downing, 2006).

Scully (2017) invalidated the perception that MCQs can only assess lower ordered thinking and Palmer EJ and Devitt (2007) illustrated that the percentage of question testing lower ordered thinking is same in both MCQs and MEQs. It also shows that a well-constructed MCQ is a better tool to assess higher ordered thinking in medical students than an MEQ ( Palmer & Devitt, 2007). There is nothing innate in the MCQ assessment format which prevents testing of higher-ordered thinking ( Norcini, Swanson, Grosso, Shea, & Webster, 1984). Besides, medical schools are training their faculty members to develop multiple-choice questions which ensure assessment of higher ordered thinking of their students. ( Vanderbilt et al., 2013).

## Conclusion

The higher ordered thinking in undergraduate medical students can be better assessed through well-constructed multiple-choice questions as compared to modified essay questions. Therefore, well-constructed MCQS should be considered a reasonable substitute for MEQs because of a variety of other advantages it provides over MEQs.

## Take Home Messages

A well-constructed MCQS should be considered a reasonable substitute for MEQs because of a variety of other advantages it provides over MEQs.

## Notes On Contributors

Dr. Arslaan Javaeed is an assistant professor of Pathology in Poonch Medical College, Rawalakot, Pakistan and is doing his masters in Health Profession Education from Faculty of Education, University of Ottawa, Ottawa, Canada.
