# Supplementary material for: Assessment of Higher Ordered Thinking in Medical Education: Multiple Choice Questions and Modified Essay Questions
Source: MedEdPublish (2016). 2018 Jun 12;7:128. [Version 1] doi: 10.15694/mep.2018.0000128.1 (PMC10699377; doi:10.15694/mep.2018.0000128.1)
Supplement: Peer review history

# Reviewer report: Reviewer response for version 1

**DOI:** 10.21956/mep.19597.r29102

**Published:** 2019-03-13

**Authors:** sathyanarayanan varadarajan

**Affiliations:** 1 SRM Medical College, SRM University

This review has been migrated. The reviewer awarded 4 stars out of 5 This interesting article is about assessing the ability of a well-constructed Multiple-Choice Question (MCQ) to assess higher order thinking skills as compared to a Modified Essay Questions (MEQ) in medical education. This has been done by the author by searching and analyzing the medical education literature available. The author has compared MEQ and MCQ with suitable Examples of multiple choice questions assessing higher order thinking i.e. application, analysis and evaluation levels. The author concludes that well-constructed MCQs should be considered as a reasonable substitute for MEQs because of a variety of advantages it provides over MEQs. But the real challenge lies in constructing MCQs appropriately that can assess the higher order thinking skills truly and effectively. This paper will be useful for all medical educators, who are involved in Evaluation and Assessment.

Reviewer Expertise:

NA

No decision status is available

# Reviewer report: Reviewer response for version 1

**DOI:** 10.21956/mep.19597.r29103

**Published:** 2018-06-24

**Authors:** Sateesh Babu Arja

**Affiliations:** 1 Avalon University School of Medicine

This review has been migrated. The reviewer awarded 4 stars out of 5 I enjoyed reading this paper. It is an interesting paper which is in favor of multiple choice questions over modified essay questions. I agree with authors it is required to see how well constructed is the assessment rather than the format of the assessment. As long as the assessments fulfill the important traits like Validity, Reliability, feasibility, educational effect, and catalytic effect, they are good practices. Validity can be determined by the Miller's pyramid, the content of the exam, and mapping with learning objectives. It is known that MCQs are clearly helpful in assessing application and analysis levels. But it is interesting to see that MCQs can be used to assess evaluation level too. This paper is good for faculty members, academic administrators, and assessment committees.

Reviewer Expertise:

NA

No decision status is available

# Reviewer report: Reviewer response for version 1

**DOI:** 10.21956/mep.19597.r29100

**Published:** 2018-06-17

**Authors:** P Ravi Shankar

**Affiliations:** 1 American International Medical University

This review has been migrated. The reviewer awarded 4 stars out of 5 The author discusses an important point in this article. He describes how a well-constructed multiple choice question (MCQ) can test higher-order skills. The author has provided some examples of MCQs testing higher order skills and has described both the original Bloom’s taxonomy and its recent revision. The article could be further strengthened by the author providing tips to construct MCQs testing higher order skills. In offshore Caribbean medical schools MCQs are widely used for assessment and are modeled after the United States Medical Licensing exam (USMLE) pattern. A problem is MCQs are the predominant method of assessment and other methods are not widely used. Many of these schools concentrate on preparing students for success in the USMLE which predominantly uses single response MCQs. MCQs are objective, unbiased and can be evaluated by machine and are widely used in various licensing and other high stakes exams. All medical educators will find this article to be of interest.

Reviewer Expertise:

NA

No decision status is available

# Reviewer report: Reviewer response for version 1

**DOI:** 10.21956/mep.19597.r29101

**Published:** 2018-06-17

**Authors:** Tayyaba Khan

**Affiliations:** 1 PMC

This review has been migrated. The reviewer awarded 5 stars out of 5 I thoroughly enjoyed reading this research article. Mostly multiple choice questions are used to assess factual knowledge only and this article indicates need of wellconstructed mcqs for their proper usage.

Reviewer Expertise:

NA

No decision status is available
